# The role of the type 7 adenylyl cyclase isoform in alcohol use disorder and depression

**DOI:** 10.3389/fphar.2022.1012013

**Published:** 2022-10-28

**Authors:** Boris Tabakoff, Paula L. Hoffman

**Affiliations:** ^1^ Department of Pharmaceutical Sciences, Skaggs School of Pharmacy and Pharmaceutical Sciences, University of Colorado Anschutz Medical Campus, Aurora, CO, United States; ^2^ Lohocla Research Corporation, Aurora, CO, United States; ^3^ Department of Pharmacology, School of Medicine, University of Colorado Anschutz Medical Campus, Aurora, CO, United States

**Keywords:** type 7 adenylyl cyclase (AC7), alcohol use disorder, depression, disease markers, medication development

## Abstract

The translation of extracellular signals to intracellular responses involves a number of signal transduction molecules. A major component of this signal transducing function is adenylyl cyclase, which produces the intracellular “second messenger,” cyclic AMP. What was initially considered as a single enzyme for cyclic AMP generation is now known to be a family of nine membrane-bound enzymes, and one cytosolic enzyme. Each member of the adenylyl cyclase family is distinguished by factors that modulate its catalytic activity, by the cell, tissue, and organ distribution of the family members, and by the physiological/behavioral functions that are subserved by particular family members. This review focuses on the Type 7 adenylyl cyclase (AC7) in terms of its catalytic characteristics and its relationship to alcohol use disorder (AUD, alcoholism), and major depressive disorder (MDD). AC7 may be part of the inherited system predisposing an individual to AUD and/or MDD in a sex-specific manner, or this enzyme may change in its expression or activity in response to the progression of disease or in response to treatment. The areas of brain expressing AC7 are related to responses to stress and evidence is available that CRF1 receptors are coupled to AC7 in the amygdala and pituitary. Interestingly, AC7 is the major form of the cyclase contained in bone marrow-derived cells of the immune system and platelets, and in microglia. AC7 is thus, poised to play an integral role in both peripheral and brain immune function thought to be etiologically involved in both AUD and MDD. Both platelet and lymphocyte adenylyl cyclase activity have been proposed as markers for AUD and MDD, as well as prognostic markers of positive response to medication for MDD. We finish with consideration of paths to medication development that may selectively modulate AC7 activity as treatments for MDD and AUD.

## Introduction

“In the beginning….”, Blenkinsopp (2011) (which for cyclic adenosine 3′-5′ monophosphate (cyclic AMP, cAMP) and adenylyl cyclase, by the Western calendar, was 1957), Sutherland and Rall identified a chemical which was produced during incubation of liver “particles” (homogenates) with ATP, magnesium, glucagon, and epinephrine (Sutherland and Rall, 1957). They reported that “similar or identical” compounds could be isolated from heart, skeletal muscle, and brain. This was the first identification of the “second messenger” (Sutherland et al., 1968) molecule formally known as adenosine 3′-5′ monophosphate or cyclic AMP. In 1962, Sutherland, Rall, and Menon described “adenyl cyclase” the enzyme that catalyzed the synthesis of cyclic AMP from ATP (Sutherland et al., 1962). It soon became obvious that adenyl cyclase (now referred to as adenylyl cyclase) and cyclic AMP were critical intermediaries in the actions of a plethora of hormones and other first messengers which interacted with their cognate receptors and modulated adenylyl cyclase activity. The receptors that can modulate adenylyl cyclase activity are within the family of G-protein-coupled receptors (GPCRs).

The discovery of guanine nucleotide binding proteins (G-proteins), which couple the GPCRs to adenylyl cyclase, is ascribed to Alfred Gilman and Martin Rodbell. Martin Rodbell showed in 1971 that the relay of a signal from a receptor on the exterior of a cell to the cell interior requires three functional units: 1) the receptor, 2) a “transducer” that utilizes GTP and, 3) an “amplifier” that generates a second messenger (Rodbell et al., 1971a; Rodbell et al., 1971b). The character of the transducer that interacts with adenylyl cyclase (the “amplifier”) was then described by the laboratory of Alfred Gilman and colleagues in 1980 (Schleifer et al., 1980). They isolated trimeric proteins (“G proteins” consisting of α, β and γ subunits) from brain that could restore coupling of receptors to adenylyl cyclase in mutant leukemia cells which lacked such proteins (Schleifer et al., 1980). Through such reconstitution, one could restore the responses of the mutant cell to hormones. Interestingly, in the Nobel Lecture by Rodbell on the occasion of the Nobel Prize to Gilman and Rodbell in 1994, it was stated, “In some common disease states the amounts of G-proteins in cells are altered. There can be too much or too little of them. In for example diabetes and in alcoholism there may be some symptoms that are due to altered signaling *via* G-proteins” (Rodbell, 1995). This statement bears some truth, but there is more to the story.

Before proceeding, one should touch on how the second messenger cyclic AMP produces its effects within the cell. The initial discovery of an effector mediating the signal initiated by intracellular levels of cyclic AMP garnered yet another Nobel prize. This prize went to Edwin G. Krebs and Edward Fischer for their discovery and characterization of protein kinase A (Krebs et al., 1964; Walsh et al., 1968), and the description of protein phosphorylation cascades that are the final mediators of much of cellular function, from energy metabolism to gene transcription, to cell survival (Ahn et al., 1991). Although it was initially thought that protein kinase A was the sole mediator of cyclic AMP action, it is now evident that cyclic AMP acts through several effectors. The three most studied effectors are: 1) protein kinase A, 2) the exchange proteins activated by cyclic AMP (Epac) (Robichaux and Cheng, 2018), and 3) cyclic nucleotide-gated ion channels (CNG channels (van der Horst et al., 2020). More recently additional cAMP effector proteins have been identified including hyperpolarizing activated cyclic nucleotide-gated potassium channels (HCN1-4) (Brand, 2019; Santoro and Shah, 2020); the Popeye domain-containing proteins (POPDC proteins) (Brand, 2019); and cyclic nucleotide receptors involved in sperm function (CRIS) (Krähling et al., 2013). In addition, some isoforms of phosphodiesterases (PDE) which degrade cAMP are also regulated allosterically by cAMP (Omori and Kotera, 2007).

In the late 1970s and early 1980s, the proposal that ethanol produced its neurobiological effects by perturbing the physical structure of neuronal membranes held dominance in the area of alcohol research. It struck us (Tabakoff and Hoffman, 1979) that the adenylyl cyclase system of brain would be a good test of how, and if, in neuronal membrane preparations, a disruption of membrane structure would translate into a perturbation of an important signal transduction system (i.e., adenylyl cyclase). By then the work of Sutherland, Rall and Gilman had demonstrated that at least three different membrane-bound protein components had to act in concert to modulate the production of cyclic AMP (this is not counting the fact that G-proteins were trimers of α, β and γ protein subunits). Thus we thought that the adenylyl cyclase system would be excellent for reflecting ethanol’s lipid perturbing properties.

This review puts in historical context the work that established that ethanol, at concentrations found in the brain of inebriated individuals, can significantly alter adenylyl cyclase activity and the adaptive responses seen in the adenylyl cyclase signaling system with chronic exposure of the brain to ethanol. The discoveries of multiple isoforms of adenylyl cyclase disclosed that one particular isoform was most sensitive to ethanol’s actions, and genetic manipulation of the expression of this isoform revealed the biological context for this isoform’s actions within particular neuronal systems, such as the GABA neurons of the amygdala and nucleus accumbens, and the corticotropes of the pituitary. The effects of genetic manipulation of this isoform on the behavioral repertoire of a genetically modified animal, indicated that alcohol consumptive behavior, and behaviors associated with animal models of MDD, were related to the levels of expression of the alcohol sensitive adenylyl cyclase in brain, but these effects were influenced by the sex of the animal. Extrapolating from mouse to human, the levels of expression of the alcohol-sensitive adenylyl cyclase in brain or cells in blood, established that measures of this enzyme’s expression or activity could be considered state or trait markers of AUD or MDD. A more recent finding, demonstrating that the alcohol-sensitive adenylyl cyclase is the primary cyclase in cells of the immune system, opens another aspect in the biology of this enzyme isoform, and its relation to ethanol’s action in humans. An added facet to this observation is the dominant presence of this isoform in microglia in brain, and the possible implications of this fact on microglial activation, and the effects of ethanol on this aspect of brain function. We finish with some observations on the prospects of isoform-specific modulators of adenylyl cyclase activity, and the possibility of their use as medications in treating AUD and MDD.

### Ethanol’s action on brain membrane-bound adenylyl cyclase

In our initial studies, we used cell membrane preparations of the striatum of mouse brain and measured the production of cyclic AMP (Luthin and Tabakoff, 1984). We found that concentrations of ethanol up to 500 mM had no effect on basal adenylyl cyclase activity. Only after the addition of Gpp (NH)p, the non-hydrolysable analog of GTP, to the assay mixture did ethanol, at concentrations as low as 50 mM, produce significant increases in production of cyclic AMP. This was the first indication that ethanol’s actions on adenylyl cyclase were related to the presence of the activated G protein in the assay, but the low ethanol concentration necessary to produce the measurable effect did not well support the hypothesis that ethanol was acting by perturbing membrane lipid structure.

We followed these studies with the examination of the acute and chronic effects of ethanol administration on dopamine-stimulated adenylyl cyclase activity in striatal membrane preparations (Tabakoff and Hoffman, 1979). Ethanol, at concentrations of 50 mM, added to striatal membrane preparations from “ethanol naive” mice increased dopamine-stimulated adenylyl cyclase activity without changing the potency of dopamine. Mice were then chronically treated with ethanol to produce physical dependence, and sacrificed at various times over the ensuing 7 days following withdrawal. We noted that after ethanol was cleared from their systems (8–24 h), the response of striatal membrane adenylyl cyclase to dopamine was reduced in a time-dependent manner. The reduction in response became evident at a time when withdrawal signs were reaching their peak, and continued to decrease through the initial 24 h of the withdrawal period. The phenomenon was reversible, with the responsiveness to dopamine beginning to return to control levels by 36 h post withdrawal. The responsiveness to exogenously added ethanol remained intact during this period. Thus, ethanol could still increase the response of adenylyl cyclase to addition of dopamine, and at particular concentrations (50 mM), produce a response to dopamine that equaled the response to dopamine of the membranes from control mice, measured without the addition of ethanol. From these studies, we surmised that the withdrawal from a chronic ethanol feeding paradigm generated the diminution of the response of striatal adenylyl cyclase to dopamine, and the reintroduction of ethanol could “normalize” this function of dopamine. Rabin and colleagues (Rabin et al., 1980; Rabin and Molinoff, 1981) followed with an attempt to replicate our results. Their work demonstrated that the addition of ethanol (≥ 68 mM) to incubations containing striatal homogenates increased dopamine stimulated adenylyl cyclase activity, but when they treated mice chronically with an ethanol-containing liquid diet to produce physical dependence, and isolated striatal membranes from control and ethanol-fed mice 24 h after withdrawal, they found no difference in response to dopamine between the preparations from the ethanol-treated and control mice (Rabin et al., 1980). Interestingly, they replicated additional aspects of our prior studies such as the increase in levels of muscarinic cholinergic receptors in the striatum of the ethanol-fed and withdrawn mice (Tabakoff et al., 1979). Further research using rats chronically treated with intraperitoneal (ip) injections of ethanol, produced ambiguous results regarding dopamine-stimulated adenylyl cyclase activity in striatum of the ethanol-treated rats during withdrawal. Seeber and Kuschinsky (1976) found that 15 h after withdrawal, there was a “slight postjunctional subsensitivity to dopamine,” but the differences were not statistically significant. One of the variables contributing to the disparate findings regarding the effects of chronic treatment with ethanol and withdrawal on dopamine-stimulated adenylyl cyclase activity in the striatum, is the time of measurement of the enzyme activity after withdrawal. Our studies demonstrated that the differences become evident after some period after withdrawal, are evident during the first 24 h, and begin to disappear by 72 h after withdrawal (Tabakoff and Hoffman, 1979). The measurements at a single timepoint during the first 24 h after withdrawal in mice (Rabin et al., 1980), or rats (Seeber and Kuschinsky, 1976), may miss the optimal time to demonstrate the changes in the striatum. Such studies that will have to be better designed in the future, including a careful preparation of the cell membranes used in the analysis. As will be discussed later, the phosphorylation state of particular isoforms of the adenylyl cyclase is important in the measure of G-protein stimulated activity. Thus, a more careful assessment of the time course of changes in striatal dopamine-stimulated adenylyl cyclase activity, and more concern about factors that may contribute to ethanol-induced changes, continue to be warranted. It should be noted that there is consistent evidence that the changes in the dopamine-stimulated adenylyl cyclase activity in the striatum are not due to changes in the D1 dopamine receptors (Tabakoff and Hoffman, 1979; Rabin et al., 1980), and probably not due to changes in the quantity of the stimulatory G-protein α subunit (Tabakoff et al., 1995).

A reason for giving emphasis to changes in dopamine function in the striatum of ethanol withdrawn animals, is the currently popular concept regarding allostasis and reward deficit that drive withdrawal-induced alcohol consumption (Koob and Volkow, 2016). If dopamine function in the striatum is compromised, and ethanol can normalize (increase) the response to dopamine effects, these factors could put biological context to the behavioral phenomena.

Receptor-activated adenylyl cyclase activity has also been measured in other brain areas of animals chronically treated with ethanol (Saito et al., 1987). Isoproterenol (β-adrenergic receptor agonist)-stimulated adenylyl cyclase activity in cerebral cortical membranes was shown to be reduced after chronic treatment of mice with ethanol. These changes were normalized within 24 h after withdrawal (Saito et al., 1987). Rabin (1990a), however, found that there were no differences in isoproterenol-stimulated adenylyl cyclase when measured 20 h after withdrawal. Again, the changes seen in the cortical tissue follow a particular time course, and studies of the changes need to include measurement at several time points after withdrawal. Interestingly, Rabin (1990a), Rabin (1990b) did find that ethanol treatment of cells (cerebellar granule cells and PC12 cells) in culture for several days decreased the maximum activation of adenylyl cyclase in these cells by isoproterenol or 2-chloroadenosine. Studies with HEL cells in cultures containing ethanol also demonstrated ethanol-dependent reduction in PGE1-stimulated adenylyl cyclase activity (Rabbani and Tabakoff, 2001). It may be concluded that a down-regulation of adenylyl cyclase activity in certain areas of brain, and in immune/platelet cell precursors, does take place in animals or cells chronically treated with ethanol, but a careful and more extensive monitoring of the time course of events is necessary to further substantiate such phenomena. Given this observation, the mechanism of this phenomenon becomes of interest.

Forskolin is a diterpene alkaloid which has been shown to bind to most forms of adenylyl cyclase, and radioactively-labeled forskolin has also been used to quantify adenylyl cyclase protein levels (Insel and Ostrom, 2003). Measurement of forskolin-stimulated adenylyl cyclase activity in cerebral cortical membranes of chronically ethanol-treated mice, demonstrated that the stimulation by forskolin had a similar potency in tissue from control and ethanol-treated mice, but the maximal effects were significantly lower in the ethanol-fed animals (Valverius et al., 1989). Autoradiographic analysis of ^3^H-forskolin binding across the various areas of mouse brain revealed differences between control and ethanol-fed animals in several brain areas (Valverius et al., 1989). It should be noted that in these studies, the animals were sacrificed while still intoxicated. Lower levels of forskolin binding were found in areas such as the cortical areas, nucleus accumbens, amygdala, hippocampus, and globus pallidus, while no significant differences were noted in the caudate putamen or cerebellum at this point in time. One can come to a conclusion that chronic ethanol administration to rodents produces diminutions in the expression of adenylyl cyclase protein in certain areas of brain, and the physiological phenomena accompanying the withdrawal from ethanol may produce changes in adenylyl cyclase activity in other brain areas.

Overall, the early studies demonstrated that ethanol’s stimulation of adenylyl cyclase activity was dependent on the presence of G protein, and chronic exposure of cells in culture to ethanol resulted in a down-regulation of GPCR-mediated activation of adenylyl cyclase. The results with brain tissue taken from animals that had been chronically treated with ethanol are somewhat ambiguous, but the ambiguity comes mainly from the fact that the diminution in GPCR-stimulated adenylyl cyclase activity after ethanol treatment and withdrawal follows a particular time course, and the phenomenon may be missed if only one timepoint after withdrawal is studied. The down-regulation of GPCR-stimulated adenylyl cyclase activity by chronic exposure of the organism to ethanol may well be related to ethanol craving and CNS hyperexcitability that occur during withdrawal.

### The identification of adenylyl cyclase isoforms and the ethanol-sensitive adenylyl cyclase

The work described to this point was performed prior to the discovery that there were multiple forms of adenylyl cyclase. Gilman’s laboratory (Krupinski et al., 1989; Tang et al., 1991) reported on the first isoform of adenylyl cyclase aptly named the Type 1 adenylyl cyclase. Soon after Type 2 adenylyl cyclase was described (Feinstein et al., 1991). Type 3 adenylyl cyclase was first described by Bakalyar and Reed (1990), Type 4 by Gao and Gilman (1991), Type 5 by Ishikawa et al. (1992), Type 6 by Katsushika et al. (1992), Yoshimura and Cooper (1992) and Krupinski et al. (1992), Type 8 by Cali et al. (1994), and Type 9 by Hacker and Storm (1998) and Premont et al. (1996). All of these were membrane-bound forms of adenylyl cyclase and there was one form of adenylyl cyclase that was found to be cytosolic (Type 10) (Buck et al., 1999). Soon after the findings regarding the Type 6 adenylyl cyclase, we isolated a sequence from human erythroleukemia (HEL) cells that showed sequence similarities to other adenylyl cyclases, but also displayed characteristic functional differences (Hellevuo et al., 1993). After cloning the full length sequence, expressing the protein, and characterizing its activity, it became clear that this adenylyl cyclase was unique and was named the Type 7 adenylyl cyclase (Hellevuo et al., 1995). At the same time, Watson et al. (1994), isolated and characterized a similar adenylyl cyclase from rat brain illustrating that the Type 7 adenylyl cyclase (AC7) was present in rodents, as well as in human tissues.

The characteristics of the discovered adenylyl cyclases were such that they could be fitted into 4 families (Devasani and Yao, 2022). AC7 joined the family containing the Type 2 and Type 4 adenylyl cyclases. This family was distinguished by its insensitivity to calcium with or without calmodulin, insensitivity to inhibition by the Gαi protein and by the stimulatory effects of phorbol esters acting through PKC, as well as the co-stimulation by the βγ subunits of the G proteins acting simultaneously with the Gαs protein (Yoshimura et al., 1996). The βγ subunits that act in concert with Gαs were found to be derived from Gi/o proteins coupled to GPCRs that are many times thought to be inhibitory to adenylyl cyclase activity (Yoshimura et al., 1996; Rhee et al., 1998). Thus, for example activation of opiate or cannabinoid receptors (coupled to Gi) simultaneously with activation of D1 dopamine receptors (coupled to Gs) further activates AC7 and the other members of its family.

We found another feature that distinguished the AC7, i.e., ethanol could stimulate the activity of AC7 to a two-to-three times greater extent than any of the other adenylyl cyclases (Yoshimura and Tabakoff, 1995; Yoshimura and Tabakoff, 1999). An activated Gαs protein was still necessary to witness this effect of ethanol (Yoshimura and Tabakoff, 1995). AC7 was also the most responsive to activation by phorbol esters, in comparison to the other members of its family (Type 2 and Type 4), and the stimulation of AC7 by phorbol esters involved the presence of an activated Gαs (Yoshimura and Cooper, 1993; Hellevuo et al., 1995). It became of interest to consider whether ethanol and phorbol esters may be utilizing a similar pathway to accomplish the activation of AC7. Phorbol esters are known activators of members of the protein kinase C(PKC) family (Reyland, 2009). There are 10 known PKCs and 8 of them are activated by phorbol esters (classical PKCs: α, β1, β2 and γ, which are responsive to phorbol esters and diacylglycerol, and are dependent on calcium binding for their activity; novel PKCs: δ, θ, ε, η which are responsive to phorbol esters and diacylglycerol but insensitive to calcium; and atypical PKCs: ζ, λ/ι, which depend on binding of phosphatidylinositol 3, 4, 5-trisphosphate or ceramide for activation (Reyland, 2009; Newton and Brognard, 2017)). All of the PKC enzymes are processed by a series of ordered phosphorylations and conformational changes to attain a catalytically active form. The enzymes are maintained in an inactive state until the binding of the proper second messenger (in the case of PKCδ, for example, the second messenger is diacylglycerol) and a conformational change leading to a catalytically active, open, form of this enzyme is then attained (Newton and Brognard, 2017).

At the time that involvement of PKC in the action of ethanol on adenylyl cyclase was being studied, little of the detail of the activation process for PKCs was known. However, through a series of studies dependent on the process of elimination, the PKC most likely to interact with AC7, and increase its activity, was found to be PKCδ (Nelson et al., 2003). Nelson et al. (2003) using HEL cells which contain predominantly AC7 (Hellevuo et al., 1993), demonstrated that PKCδ could phosphorylate AC7 protein, with the likely site of phosphorylation being the C1b domain of AC7 (Nelson et al., 2003).

The catalytic conversion of ATP to cyclic AMP by adenylyl cyclases involves the juxtaposition of two domains of the enzyme protein. The C1a region of the intracellular loop between the membrane spanning domains, M1 and M2 has to align with the C2 region of the C terminal tail of the adenylyl cyclase protein to form the catalytic domain. The addition of Gsα activated by GTPγS to a mixture of the C1a region peptide of the Type 1 adenylyl cyclase and the C2 region peptide of the Type 2 adenylyl cyclase increased the enzymatic activity of this mixture (cyclic AMP production) well over a thousand-fold (Yan et al., 1996). The explanation for this increase in enzymatic activity is that the activated Gsα acts as a link between the two adenylyl cyclase fragments and aligns them into the proper conformation for catalysis. Analysis of the crystal structure of the C1a and C2 regions of adenylyl cyclase in combination with Gαs and forskolin demonstrated the binding of Gαs to the C2 region and interaction with C1a region resulting in a change in orientation of these regions to each other with the resultant increase in catalytic activity (Tesmer et al., 1997).

Interposed between the C1a region and the M2 transmembrane domains is a region referred to as C1b and this region has been considered to be important for conferring isoform-specific regulatory properties to members of the adenylyl cyclase family (see references in Beeler et al. (2004)). A particularly interesting function of the C1b region is to modulate the ability of activated Gsα to promote the catalytic function of the C1a•C2 dimers (Scholich et al., 1997). Beeler et al. (2004) generated a recombinant protein representing the C1b region from AC7 (aa 506–584) and examined its effects on the catalytic function of the mixture of C1a and C2 regions from AC7. It was found that the C1b peptide inhibited the activation by Gαs of the mixture of C1a and C2 peptides derived from the AC7. The inhibition was only evident at the lower concentrations of Gαs and no effect was evident at higher concentrations (>2 μM) Gαs.

The C1b region can be phosphorylated by PKC. Shen et al. (2012) demonstrated the phosphorylation of serine 490 and 543 in the C1b region of the Type 2 adenylyl cyclase (AC2) by PKC with resultant changes in response of the enzyme to Gαi and βγ. In the AC7 sequence, several PKC phosphorylation sites are evident in the C1b region, but serines 505 and 536 are most interesting since they exist in an area of alignment to a putative binding region for PKCδ on SRBC protein (a PKCδ binding protein) and phosphorylation of these serines by PKCδ (Izumi et al., 1997) in the SRBC protein has been demonstrated. The phosphorylation of serines 505 and 536 may well allow for a more productive interaction between the C1a and C2 domains.

**FIGURE 1:**
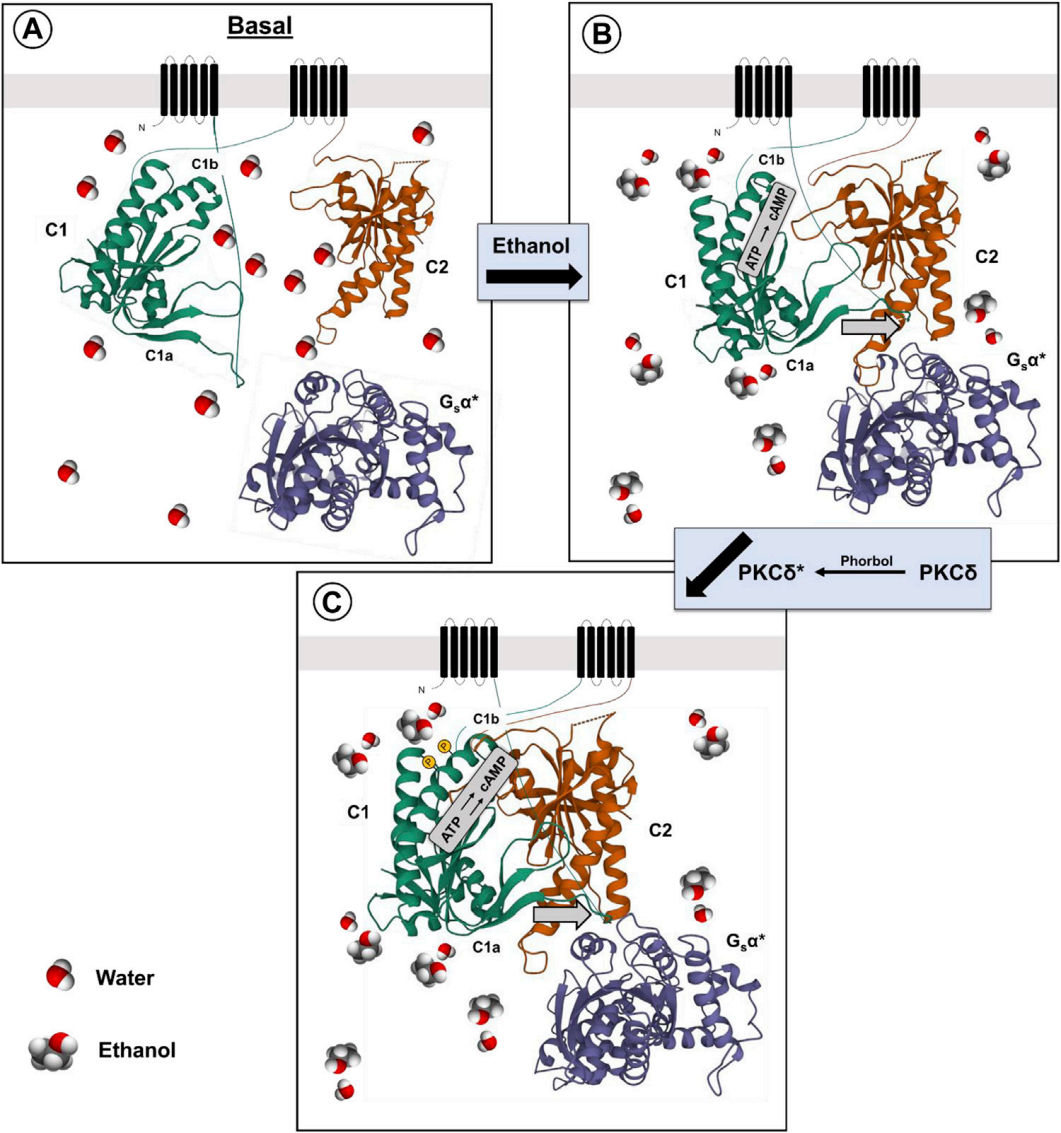
Proposed mechanism for ethanol potentiation of Gαs-mediated activation of AC7 **(A,B)**. The presence of ethanol in the water layer immediately adjacent to the C1a, C1b and C2 domains (Bagchi, 2005) promotes the alignment of these domains and interaction with Gαs (Yan et al., 1996) **(C)**. Further modification of the secondary or tertiary structure of the C1b domain can promote access to this region by PKCδ (protein•protein interaction) and phosphorylation of AC7, further enhancing Gαs-mediated activation of AC7 enzyme activity.

One can speculate that the effect of ethanol on the activity of AC7 is mediated by phosphorylation of serines in the C1b region of the AC7 enzyme. The phosphorylation could reduce the inhibition of high affinity Gαs binding by the C1b region, resulting in a greater catalytic response of AC7 to binding of Gαs in the presence of ethanol (Figure 1).

There is a caveat to this explanation of ethanol’s actions on AC7. Yoshimura et al. (2006) produced chimeras of different regions of AC2 and AC7. When expressed in HEK 293 cells, which were also transfected with dopamine (D_1_) receptors, the chimera containing the C1b and M2 region of AC7 with the C1a and C terminal region (C2) of AC2 responded to ethanol potentiation of dopamine-stimulated activity as would AC2, while the chimera containing the C1b and M2 region of AC2 with the C1a and C2 region of AC7 responded to ethanol as would be expected for AC7 (3–4 times greater response). These results led Yoshimura et al. (2006) to conclude that the C1b region of AC7 was not important for ethanol’s action. This conclusion omits consideration of the fact that ethanol’s actions on AC7 are dependent on the presence of the activated form of Gαs and that the effects of the C1b region are not independent of the other domains of the adenylyl cyclase protein. The effect of the AC7 C1b may well be tuned to the specific sequences of the C1a and C2 regions which bind Gαs in particular adenylyl cyclases (Beeler et al., 2004; Shen et al., 2012). It was notable that the chimeras in which the C1b regions of AC7 and AC2 were combined with heterologous C1a and C2 regions of these enzymes had significantly lower (5–8 times lower) dopamine-stimulated activity than in their homologous environment, and the activity in the presence of ethanol was also low. In the end, Yoshimura et al. (2006) concluded that ethanol directly influences the interaction of C1a and C2 regions of particular adenylyl cyclases, but the mechanism of this effect was left open. The control of Gαs binding by phosphorylation of the C1b region of particular adenylyl cyclases, i.e., AC7, thus remains an attractive hypothesis.

Finally, it should be noted that there is evidence that ethanol is not simply activating PKCδ to produce its effects on AC7. Rabbani et al. (1999) utilized HEL cells which naturally express AC7 to demonstrate that the effects of phorbol esters and ethanol were additive even though both the ethanol and phorbol ester effects were blocked by PKC inhibitors. This brings forth the possibility that the phorbol esters and ethanol act in a complementary manner with phorbol esters activating PKCδ, and ethanol enhancing the phosphorylation of particular substrates such as AC7. The C1b region of AC7 may be particularly sensitive to ethanol’s amphiphilic properties (Klemm, 1998) which could influence the secondary or tertiary structure of the C1b region (Beeler et al., 2004), and allow phosphorylation of serines in that region (Figure 1).

At this point, the best characterization of ethanol’s action on AC7 is that it acts as a “conditional” stimulus, with its actions dependent on the presence of the activated Gαs, and additional work needs to be performed to clarify the molecular events attendant to ethanol’s potentiation of Gαs activating properties. Ethanol’s actions on AC7 activity are evident at concentrations of 50 mM (230 mg%) or higher in cell systems in which AC7 is naturally expressed (e.g., HEL cells), and such pharmacological considerations should be applied when evaluating the physiological implications of the effects of ethanol on adenylyl cyclase-related events. It can be noted that blood alcohol levels of 200 mg% and over are not unusual for individuals coming to emergency rooms or even driving (Maruschak, 1999; Allely et al., 2006). An important issue to consider when one is evaluating the physiological impact of the effects of ethanol on adenylyl cyclase activity is the fact that adenylyl cyclases exist in “microdomains” within a cell and it is the local concentration of cAMP that instigates the downstream consequences (Zaccolo et al., 2021). At this time, the effects of ethanol on levels of cAMP have not taken this fact into account, and the changes in cAMP concentrations have been measured on a whole cell level or within an incubation volume. Localized, and possibly quite significant effects of ethanol may be diluted by such experimental approaches.

### Genetic manipulation of AC7 and the neurobiological phenotype

The generation of AC7 transgenic (TG) and heterozygous (HET) knock- down mice (Yoshimura et al., 2000; Hines et al., 2006), allowed for the qualitative assessment of the behavioral and physiological effects of AC7. The transgene used for generating the TG mice was the human form of AC7 under the control of a synapsin promoter (Yoshimura et al., 2000), while the HET mice were generated by homologous recombination with the deletion of exon 3 of AC7 (Hines et al., 2006). We were not able to produce the homozygous knock-out because the fetuses bearing the homozygous deletion died *in utero* on GD11 (Hines et al., 2006). The phenomenon of the fetus bearing two copies of the disrupted AC7 gene dying *in utero* was also noted more recently by Duan et al. (2010), highlighting the importance of AC7 in development. The initial choice of the biological systems, and then the behaviors to be examined, were based on the known involvement of adenylyl cyclase as an effector for dopamine D1 and D2 receptors and the corticotropin-releasing factor (CRF) receptors. An elegant addition to the work on involvement of AC7 in the functions of CRF in brain and pituitary, was the work of Duan et al. (2010) who used genetic manipulation of AC7 expression in cells of the peripheral immune system to demonstrate that AC7 was integral to the innate and adaptive responses of the immune system.

#### AC7 and CRF receptor coupling in the amygdala

CRF acting within the amygdala has been linked to depression and anxiety disorders in humans (Binder and Nemeroff, 2010), and to anxiety-like, and alcohol consumptive behaviors in rodents (Agoglia and Herman, 2018). CRF and CRF1 receptors also appear to be involved in alcohol withdrawal-induced anxiety and increased alcohol consumption in alcohol-dependent animals after withdrawal (craving?) (Agoglia and Herman, 2018). Marissa Roberto and her colleagues have examined the effects of ethanol on CRF-sensitive neurons in the central amygdala (CeA) (Roberto et al., 2021). CRF acting through the CRF1 receptor, which is coupled to Gs protein, can increase GABA release, and activate post synaptic GABA-A receptors. The increased release of GABA can be measured by the increases in inhibitory post-synaptic potentials (IPSPs) (Roberto et al., 2021). Ethanol or CRF added to the CeA slice preparations were shown to significantly increase the GABA-mediated IPSPs (Roberto et al., 2021). Using the CeA slices from the WT and AC7 HET mice, Cruz et al. (2011), showed that the IPSPs measured in the presence of CRF or ethanol were reduced or absent, respectively, in the preparations from the HET mice compared to the WT mice (Cruz et al., 2011). This led to the suggestion that AC7, which in part is located presynaptically (Mons et al., 1998), can be involved in the signaling initiated by the CRF1 receptor and culminating in release of GABA. There is prior evidence that CRF1 receptors couple to both AC7 and Type 9 adenylyl cyclase (Antoni et al., 2003), and the significant diminution of AC7 in brains of the HET knock-down mice may be responsible for the reduced effects of CRF and ethanol in the CeA slice preparations. Work by Bajo et al. (2008), had demonstrated that PKCε was also involved in CRF1 receptor-mediated and ethanol-potentiated GABA release in slices of the CeA. Recording of “basal” IPSP activity attributed to spontaneous GABA release was significantly increased in tissue from animals whose PKCε was disrupted by homologous recombination (PKCε^−/−^) (Khasar et al., 1999). Additionally, the CRF1 receptor- mediated enhancement of GABA release, as well as ethanol-mediated GABA release in the CeA slices, was blocked in tissue from the PKCε^−/−^ mice (Bajo et al., 2008). There is a significant difference in the results obtained from AC7 HET mouse tissue *versus* the tissue from the PKCε^−/−^ mice (Bajo et al., 2008; Cruz et al., 2011). The basal GABA release in the slices of the PKCε^−/−^ mice was substantially increased, and thus the stores available for release by CRF or ethanol may have been depleted. In AC7 HET mice, there was no change in the basal release of GABA and thus an explanation based on depletion of GABA stores would not resonate with reduced effects of CRF and ethanol in the HET mice. The evidence for mechanistic differences in PKCε effects and the effects of AC7, thus do not contradict the evidence for PKCδ mediation of the interaction of Gαs and AC7 whether induced by receptor activation or by ethanol.

**FIGURE 2:**
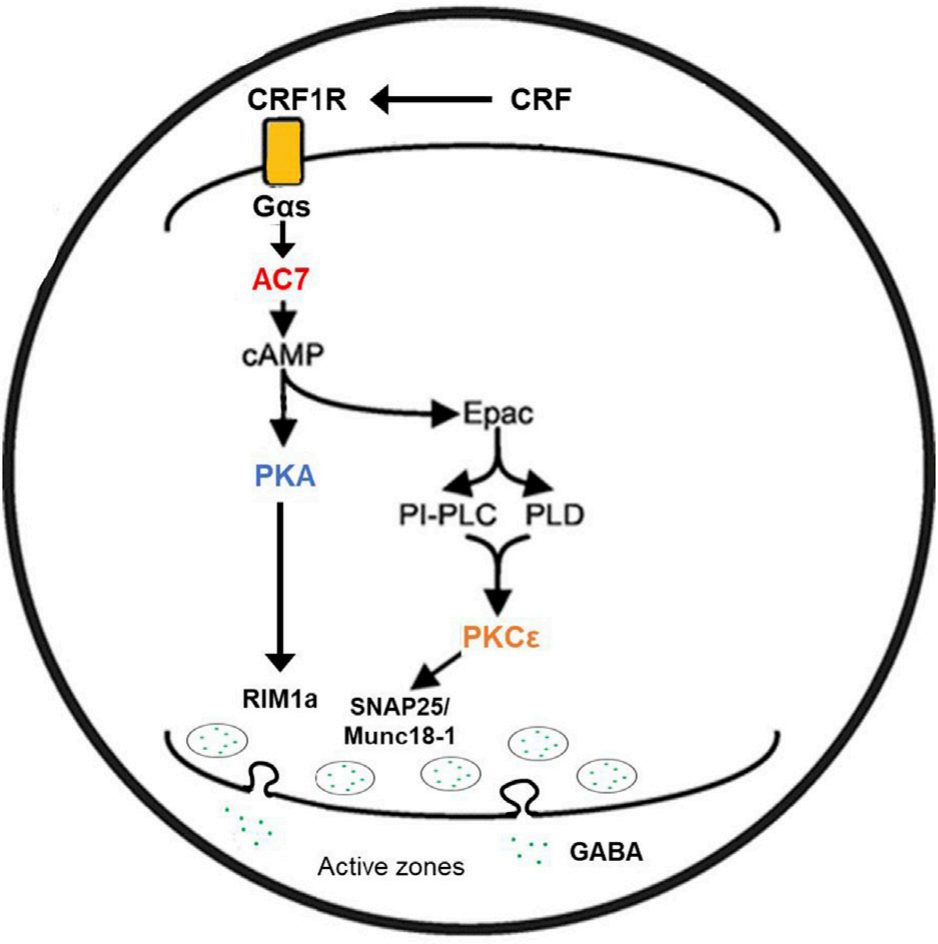
Proposed mechanism of ethanol potentiation of CRF-mediated GABA release in the central amygdala. In the pre-synaptic terminal CRF interacting with CRF1 receptors coupled to Gs protein acts to enhance the activity of AC7. Ethanol potentiates the Gαs-mediated catalytic activity of AC7 and increases the generation of cAMP. cAMP can interact with two effector molecules, PKA and Epac (Hucho et al., 2005; Robichaux and Cheng, 2018; Wang et al., 2022a) to engage two distinct pathways (Gekel and Neher, 2008) modulating transmitter release (e.g., GABA). The exocytosis of the contents of vesicles requires fusion of the vesicle with the pre-synaptic membrane and positioning of the vesicle in proximity to N or P/Q-type Ca^++^ channels (Südhof and Rizo, 2011). cAMP has also been shown to modulate vesicle loading with neurotransmitter (Costa et al., 2017). The fusion of vesicles with the synaptic membrane, pore formation, and transmitter release requires the interaction of several proteins including RIM proteins, Munc 13–1, Munc 18–1, and SNAP proteins. These proteins can be phosphorylated by PKA or PKCε (Lonart et al., 2003; Sossin, 2007; Brockmann et al., 2020) and such phosphorylation modifies their function. The change in function of the transmitter release machinery can be measured by amplitude and frequency of post-synaptic mIPSPs or mEPSPs. Ethanol-induced potentiation of CRF-initiated GABA release in the central amygdala (Nie et al., 2009) was diminished by knockdown of AC7 or knockout of PKCε (see text), and this illustration provides a rendition of the interactive pathways by which the observed effects can be explained.

A parsimonious reconciliation (Figure 2) of the involvement of both PKCε and the adenylyl cyclase system can be considered by invoking a cAMP to PKCε communication link. Such a link has already been established for excitatory transmitter release in the CNS (Gekel and Neher, 2008). Hucho et al. (2005) presented evidence that Epac is central for the activation and translocation of PKCε in neurons of the dorsal root ganglion, and that adenylyl cyclase activation *via* Gαs is the initiator of this cascade. Wang et al. (2022b) further elucidated the role of Epac-PKCε in the facilitation of docking and release of the contents of synaptic vesicles in parallel fibers of the cerebellum. If similar events are evident in GABAergic neurons (Robichaux and Cheng, 2018), then two related pathways (adenylyl cyclase/cAMP/PKA or PKCε-mediated) or one sequential pathway (adenylyl cyclase, Epac, PKC) could explain the effects of both AC7 and PKCε on modulation of CRF-mediated GABA release by ethanol.

#### AC7 and CRF receptor coupling in the pituitary

**FIGURE 3:**
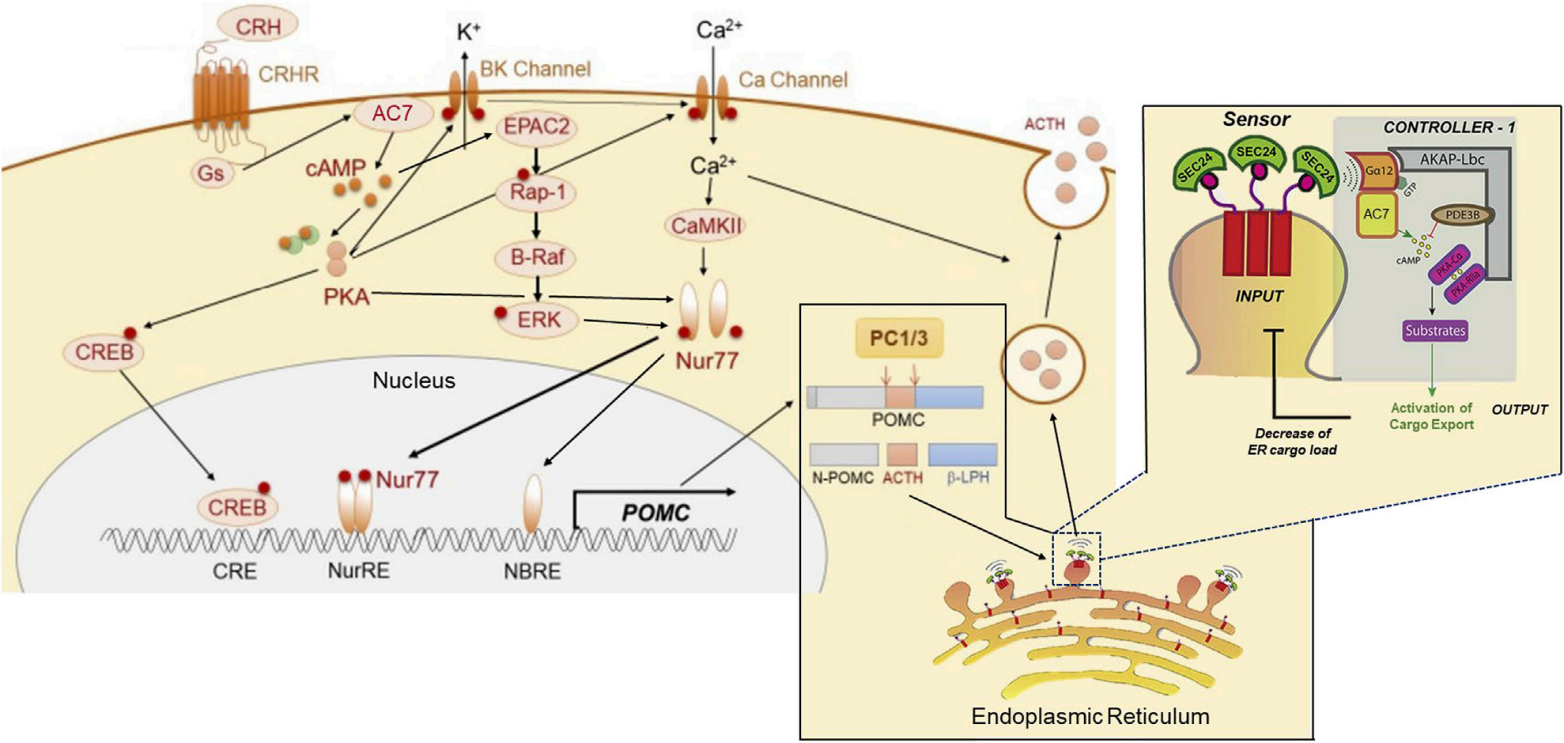
Proposed dual sites of action of AC7 in peptide (ACTH) synthesis and release. AC7 can participate in both the control of synthesis and release of ACTH. CRF (CRH) activates AC7 through a Gs-coupled mechanism and increases production of cAMP. The cAMP enhances the active state of both PKA and Epac2. PKA acts through its canonical CREB transcription activator pathway by phosphorylating CREB and translocating it to the nucleus to bind to its DNA promoter sequence. The binding of CREB to DNA is necessary but not sufficient to activate the transcription of POMC (the precursor to ACTH). The activation and DNA binding of Nur77 is a requirement for initiation of transcription. The phosphorylation and translocation of Nur77 to the nucleus, requires a coordinated interaction of ERK, PKA and CAMKII (Kovalovsky et al., 2002; Zhang and Heaney, 2020). Two sites for Nur77-binding exist upstream of the POMC transcription initiation site, a Nur77-binding response element (NBRE), and a Nur response element (NurRE), and both need to be occupied by Nur77 to initiate the transcription of POMC. Once the POMC RNA is produced, it is translocated to the endoplasmic reticulum for protein synthesis, and the processing of the pre-propeptide (POMC) by proprotein convertase (PC1/3) into ACTH and the other POMC derived peptides. The ACTH has to be loaded into dense core vesicles which are generated by “budding” of the endoplasmic reticulum, and the “cargo” of these vesicles (ACTH) is then readied for release by a calcium-dependent mechanism through fusion with the cellular membrane. AC7 is integral in the process of loading and preparing vesicles for release. AC7 is located on the endoplasmic reticulum membrane where it can be activated by Gαq12. The activation of Gq12 by guanine nucleotide exchange is instigated by a sensor on the budding vesicle, SEC24. The cAMP produced by AC7 at this site can activate PKA (which is recruited to this site by an AKAP). PKA-dependent phosphorylation of an ill-defined substrate is then central to the process of release of the concentrated cargo by controlling the uncoating and separation of the maturing vesicles from the endoplasmic reticulum. This Figure was generated based on illustrations and information provided by Fukuoka et al. (2020), and Subramanian et al. (2019).

The effects of ethanol on CRF-mediated signaling (Figure 3) have been further investigated using CRF-mediated ACTH release in the pituitary of the HET knock-down and TG mice overexpressing AC7. Assessment of the forms of adenylyl cyclase present in the mouse pituitary indicated the presence of the Type 2, Type 3, Type 6, and Type 7 (Pronko et al., 2010). It should be noted that Type 9 adenylyl cyclase has been reported to be present in rodent corticotropes (Antoni et al., 2003), but was not found in the mouse pituitary using microarray analysis (Pronko et al., 2010).

In the WT, AC7 HET, and AC7 TG mice (Pronko et al., 2010), the most profound differences were noted in the plasma ACTH levels of the male and female mice after injection of ethanol (Pronko et al., 2010). Significant quantitative differences among WT, HET and TG mice were found in both the peak levels and AUC of the ACTH responses to injection of ethanol (these values well surpassed the levels seen after saline injection). The levels of corticosterone correlated in magnitude with plasma ACTH levels after ethanol injection. The rank order of the plasma ACTH and corticosterone levels after ethanol injection was AC7 HET < WT < TG. In all cases, female mice had higher levels of corticosterone than the males of that genotype (Pronko et al., 2010). The results of these studies again establish AC7 as an important component of the link between the CRF1 receptor, and the downstream consequences of its activation, but the differences in the corticosterone response between males and females are not explained by differences between sexes in expression of AC7 in the pituitary. The genetic manipulation of the Adcy7 gene produced similar levels of AC7 RNA in the pituitary of the male and female mice of the HET or TG genotypes (Pronko et al., 2010), and the protein levels for AC7 followed the same pattern as the RNA levels with no statistically significant differences between males and females (Pronko et al., 2010). In the WT and AC7 HET mice, the higher levels of corticosterone in females may reflect higher ACTH levels. However, ACTH levels did not differ significantly between AC TG male and female mice. One explanation of the lack of sex differences in AC7 in the pituitary, but significant sex differences in the corticosterone response of AC7 TG mice, is the observation that the adrenal tissue of females may be more responsive to ACTH than that of males (Rao and Androulakis, 2017), and at a particular level of ACTH more corticosterone would be released from the adrenals of females.

The probable involvement of AC7 in the CRF1 receptor-mediated release of ACTH brings into further consideration the importance of microdomains in the actions of ethanol on AC7. AC7 is part of what was referred to as a “signalosome” consisting of Gα_12_, AC7, PDE3B, PKA and other kinases organized around AKAP13 on the endoplasmic reticulum (Zaccolo et al., 2021). This type of signalosome has been shown to be important in the regulation of secretory function of the endoplasmic reticulum for recently synthesized and properly folded proteins (Subramanian et al., 2019). The presence of Gα_12_ in this signalosome complex is consistent with the presence of AC7 since Jiang et al. (2008) demonstrated that AC7 is a specific downstream target of the Gα_12/13_ subunits that produce an increase in AC7 activity (Figure 3).

The involvement of the “ethanol sensitive” AC7 in the ACTH/corticosterone response to ethanol administered *in vivo*, helps explain a seeming enigma with regard to responses to imbibed ethanol. Ethanol is considered an anxiolytic drug, but several reports have provided evidence that ethanol ingestion generates an increase in the circulating levels of cortisol (stress hormone) in humans. Since the anxiolytic and the cortisol elevating effects of ethanol can arise by different mechanisms and involve different areas of brain (Pronko et al., 2010; Olsen and Liang, 2017), these results can be quite compatible.

#### AC7 mediation of dopamine effects on DARPP-32 and ethanol’s actions

**FIGURE 4:**
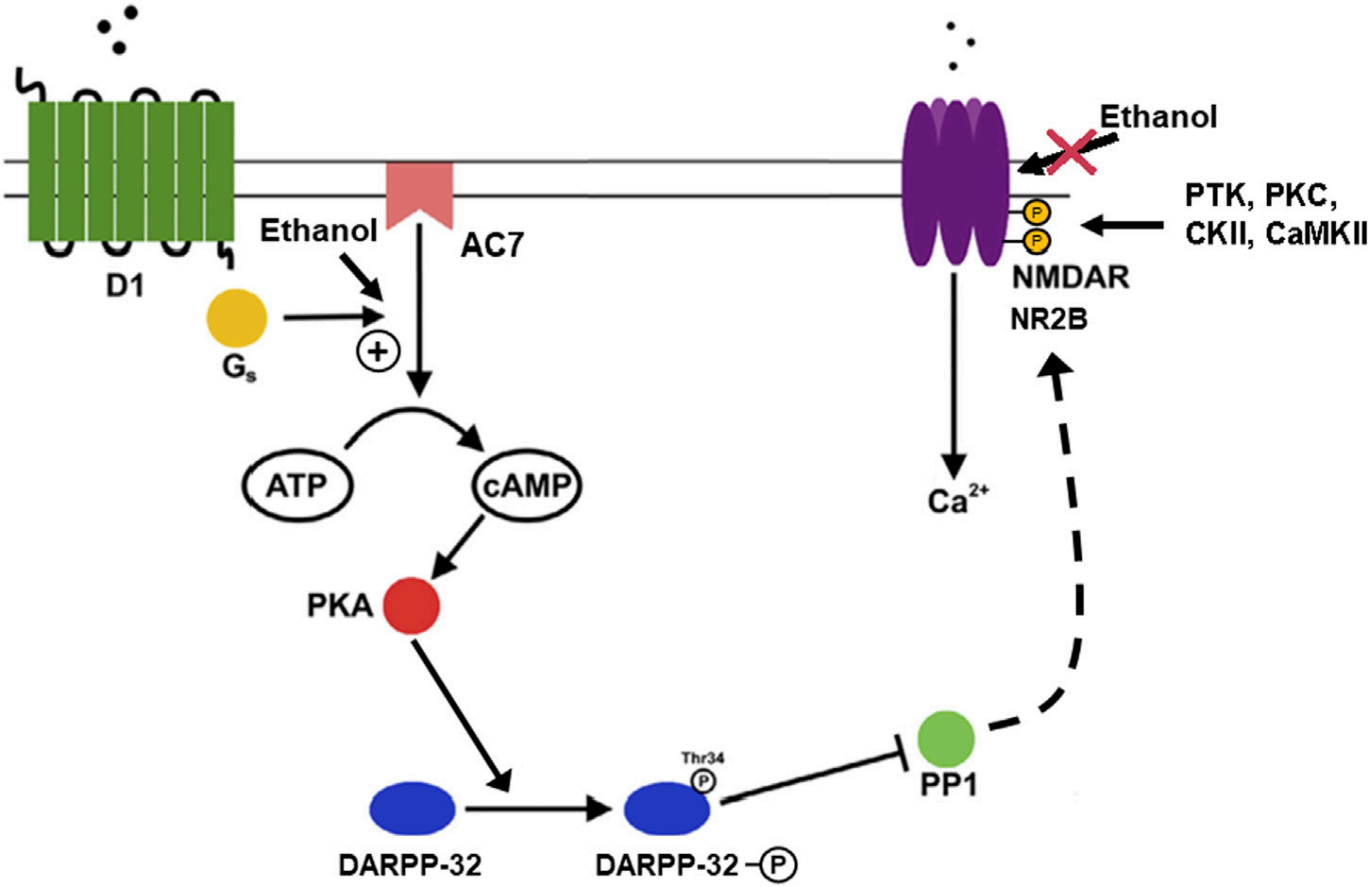
Phosphorylation of DARPP-32 mediated by dopamine through AC7 and actions of ethanol. The activity of medium spiny neurons (GABAergic neurons) in the nucleus accumbens and other areas of the striatum are important in the control of reward-related dopamine signals mediated *via* D1 and D2 dopamine receptors, with D1 receptors acting to enhance dendritic excitability (Surmeier et al., 2007). The increase in dendritic excitability is proposed to be dependent on modulation of cortico-striatal glutamatergic signaling *via* NMDA receptors located on the dendrites which also contain the D1 receptors. The link between the dopamine and glutamate signals is provided by the actions of PKA, DARPP-32, and protein phosphatase 1 (PP1) (Fernandez et al., 2006). The figure illustrates that ethanol, by acting on AC7, increases the production of cAMP which activates PKA, which in turn phosphorylates DARPP-32 on threonine 34. When phosphorylated on threonine 34, DARPP acts as an inhibitor of PP1, preventing dephosphorylation of the PP1 substrates. One of the PP1 substrates is the NMDA receptor which is a substrate for phosphorylation by protein tyrosine kinases (PTK), PKC, casein kinase (CKII), and calcium calmodulin kinase (CaMKII) (Chen and Roche, 2007). The NMDA receptor/channel is more active in its phosphorylated state (Nakazawa et al., 2001), and also resistant to the inhibitory effect of ethanol on the NMDA receptor/channel (Hoffman et al., 1989; Miyakawa et al., 1997). The overall result of these events is the sparing of the NMDA receptor from the inhibitory effects of ethanol in particular neuronal populations (Yaka et al., 2003). The implication is that ethanol can depress excitatory glutamatergic activity in certain types of cells and brain areas, but the reward-related neurons of the striatum would be spared from this inhibitory effect of ethanol by the action of DARPP-32 *via* PP1.

The DARPP-32 signaling pathway has been proposed as a therapeutic target for AUD medication development (Greener and Storr, 2022). The phosphorylation of glutamate receptors (NMDA, AMPA) on the medium spiny neurons of the nucleus accumbens is the major event which controls the strength of excitatory input to these neurons. The actions of DARPP-32 are integral in controlling the phosphorylation state of NMDA and AMPA receptors and DARPP-32 function is itself controlled by phosphorylation/dephosphorylation events (Nishi et al., 2005). The medium spiny neurons of the nucleus accumbens are the integrators of dopaminergic signals from the ventral tegmentum and glutamatergic signals from the pre-frontal cortex (Figure 4) and play an important role in mediating the reinforcing/rewarding effects of addictive drugs (Surmeier et al., 2007; Allichon et al., 2021). An important component of this integration is dopamine D1 receptor-mediated generation of cyclic AMP, the activation of PKA, the phosphorylation of DARPP on residue threonine 34 (T34 Phospho-DARPP), the inhibition of protein phosphatase-1, and the maintenance of the ionotropic glutamate receptors in their phosphorylated state (see Figure 4). Donohue et al. (2005) used the AC7 TG mice to study the phosphorylation of the DARPP-32 protein on the threonine-34 residue in the nucleus accumbens, caudate/putamen, and amygdala. The effects of ethanol administered *in vivo* on tissue obtained from these brain areas were also examined. In the brains of AC7 TG mice and WT mice, no differences in total levels of the DARPP-32 protein were evident in any of the tested brain areas (Donohue et al., 2005). In the amygdala and caudate/putamen of saline-treated (control) WT mice, the levels of T34 Phospho-DARPP were significantly lower than those in the saline-treated AC7 TG mice. Interestingly, just the opposite was true in the nucleus accumbens. The acute administration of ethanol, *in vivo*, increased the levels of T34-Phospho-DARPP in all brain areas of the WT mice. But, only in the amygdala was the effect of the transgene evident. In the amygdala of the TG mice, the administration of alcohol significantly increased the levels of T34-Phospho-DARPP beyond those produced by saline or by the same dose of ethanol in WT mice. A somewhat similar experiment was performed by Bjork et al. (2010), in which ethanol was administered to C57BL/6 mice and levels of T32-Phospho-DARPP were measured in the “striatum,” an area including the nucleus accumbens and amygdala. Ethanol administration produced a “robust” increase in T32-Phospho-DARPP which was blocked by the dopamine D_2_ receptor antagonist, sulpiride. The effect of ethanol was also blocked by administration of naloxone given prior to the administration of ethanol. The results with the dopamine D_2_ receptor antagonist, and the opiate receptor antagonist naloxone, do indicate more complexity to the phosphorylation of DARPP in the “striatum” than a simple activation of the D_1_ dopamine receptor to initiate the phosphorylation cascade. A possibility not considered by Bjork et al. (2010) was that the presence of AC7 in the “striatum” would offer the opportunity for activation of dopamine D2 or opiate receptors to potentiate the activity of AC7 through release of βγ subunits from the Gi/Go trimers (Yoshimura et al., 1996). Naloxone administration would block the opiate/D_1_ dopamine receptor additive effect (*via* Gsα and βγ) on AC7.

The contention that opiates are acting in the striatum by coupling to Gi/Go proteins to release βγ, and produce additional activation of AC7, is further supported by the work of Karlsson et al. (2016). In this work, the role of melanin-concentrating hormone in modulating ethanol-induced conditioned place preference (CPP) was investigated. This included measurement of DARPP-32 phosphorylation in WT and MCH1 receptor knock-out mice. Administration of ethanol produced a significant increase in T32-Phospho-DARPP in the shell region of the nucleus accumbens in the WT mice, but not in the MCH1 knock-out mice. The MCH1 knock-out mice also showed diminished propensity to develop ethanol-induced CPP compared to WT mice. The results seen with mice carrying the deletion of the MCH1 gene could be replicated in WT mice by the use of a MCH1 receptor antagonist (Karlsson et al., 2016). An important consideration in interpreting these results is that the MCH1 receptor is a Gi/Go-coupled receptor (Lembo et al., 1999), which is co-expressed with dopamine receptors on medium spiny neurons (Zhang et al., 2006). Again, the generation of βγ subunits upon activation of the MCH1 receptor, could act in concert with Gsα to produce an accentuated activation of AC7, and more robust generation of cAMP.

An interesting conclusion can arise from data on ethanol’s effects on DARPP phosphorylation, as well as the above-described studies on CRF-mediated GABA release in the central amygdala. One can speculate that, in neurons of the limbic system, AC7, which is responsive to both ethanol and to βγ subunits, would be the mediator of ethanol and βγ subunit effects on phosphorylation cascades in these neurons. The PKA- and Epac-mediated events downstream of the activity of AC7 would set the tone for both metabolic and neurotransmission functions in these neurons.

#### AC7 in the immune system

Duan and colleagues (Duan et al., 2010) bred mice in which one allele of the Adcy7 gene was disrupted, and although most of the offspring that were double mutants (AC7 ^−/−^) died *in utero*, approximately 2–3% survived through birth. The bone marrow from these AC7 knockout animals and bone marrow from their wild type littermates was isolated and transplanted into mice whose immune system had been destroyed by irradiation. The immune system of the recipient mice fully regenerated, producing chimeric mice bearing the donor bone marrow cells. The total number of splenocytes was reduced by more than half in the chimeric mice generated from the bone marrow of the AC7^−/−^ mice, indicating the importance of AC7 in proliferation of both B and T lymphocytes. On the other hand, when challenged with LPS, the chimeric mice with the AC7^−/−^ bone marrow generated a 3–4 times greater TNFα response compared to the mice which received the wild type bone marrow. LPS was also more lethal in the mice carrying the AC7-deficient bone marrow. Macrophages from the AC7-deficient mice produced significantly higher levels of TNFα when challenged with LPS *in vitro*, compared to mice carrying the wild type bone marrow (this response also involved yet-to-be-identified serum factors). Duan et al. (2010) also demonstrated that AC7 was necessary for an optimal antibody response when mice were exposed to antigens. The deficiency in the AC7^−/−^ chimeric mice was primarily due to AC7-dependent function of the T helper cells, even though B cell function was also disrupted in the animals with AC7^−/−^ bone marrow. In conclusion, Duan et al. (2010) state: “…AC7 is the key AC isoform in mediating cAMP response and its downstream physiological functions in the immune system”. Table 1 summarizes the neurobiological phenotypes elucidated in mice in which the expression of AC7 was manipulated.

**TABLE 1:** Phenotypes of WT, AC7 HET, and AC7 TG mice.

Behavioral phenotype			Neurobiological phenotype									
I. Alcohol consumption and preference			I. CRF- and ethanol-induced GABA release (IPSP) in CeA*									

	Alcohol consumption	Alcohol preference	WT (CRF)		, 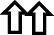							
AC7 HET (C57) vs. WT	=	=	AC7 HET (CRF)		, 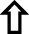							
AC7 HET (129SvEv) vs. WT	M =	=	WT (EtOH)		, 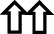							
	F, 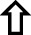	, 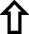	AC7 HET (EtOH)		NC							
II. Immobility in forced swim test (Depression)			II. Ethanol-mediated ACTH release (AUC)									
AC7 HET vs. WT	M =					HET		WT				TG
	F, 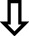		M			, 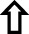		, 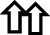				, 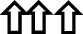
AC7 TG vs. WT	=											
	F, 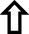		F			, 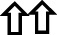		, 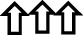				, 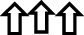
III. Immobility in tail suspension test (Depression)			III. Ethanol-mediated increase (vs. Saline) in T34 pDARPP*									
AC7 HET vs WT	M =		NucAcc			Caudate			Amygdala		
	F =										
AC7 TG vs WT	M =		WT	AC7 TG		WT	AC7 TG		WT	AC7 TG		
	F, 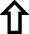		, 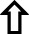	, 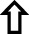		, 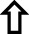	=		, 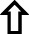	, 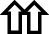		
				IV. Immune system function					
						Innate (Macrophages, TNFα)		Adaptive (Antibodies, B Cell/T-helper)	
				AC7^−/−^ Chimera vs. WT Chimera^**^		, 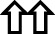		, 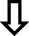	

,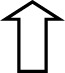, Increase ,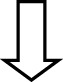, Decrease =, No Difference NC, No Change *, All males **, Chimeric mice carry WT or AC7/bone marrow

### Genetic manipulation of the type 7 adenylyl cyclase and the behavioral phenotype

Given the electrophysiological, neurochemical, and physiological results of studies with the HET, WT, and TG AC7 mice, these animals were used for behavioral measures of ethanol consumption and measures of anxiety-like and depressive phenotypes.

In measures of alcohol consumption and preference, AC7 HET mice on two genetic backgrounds (C57BL/6 and 129/SvEv) were used. C57BL/6 mice normally show a high preference for alcohol-containing solutions, and using the HET mice on the C57BL/6 background, no statistically significant differences were found between the HET and WT mice in the quantities of ethanol consumed by males or females. When AC7 was knocked down in the 129/SvEv strain, which drinks low to moderate amounts of ethanol, the females of the HET genotype consumed more ethanol, particularly at the higher concentrations of 10 and 20%. This increase in ethanol consumption resulted in a higher calculated “preference” for ethanol when water intake was taken into account (Desrivières et al., 2011). In the male HET mice on the 129/SvEv background, the amount of ethanol consumed at the highest concentration was actually less than that consumed by the WT mice, and there was no statistically significant change in the preference measure (Desrivières et al., 2011). The measure of what is called “preference” has an important concept attached. A “preference” ratio of 0.5 indicates neutrality of choice between the alternatives of the ethanol solution or plain water, while a preference ratio above 0.5 indicates a greater desire for the ethanol solution, and a preference ratio of less than 0.5 indicates an aversion to the ethanol solution. In the case of the HET female mice on the C57BL/6 background and their corresponding WT littermates, all preference ratios were above 0.8, irrespective of the status of AC7 (even though there was an evident increase to almost 1.0 in the HET mice at the lower ethanol concentrations). In the WT females of the 129/SvEv background, the “preference” for the 10 or 20% ethanol solutions was approximately 0.15 and in the HET females the ratio increased to 0.3. These results can be interpreted as indicating that on the 129/SvEv background, the diminution of AC7 in female mice, diminishes the aversion to consuming ethanol solutions containing the higher concentrations of ethanol.

Measures of behavior which is interpreted as “depressive” or “learned helplessness” were also performed in HET, WT, and TG AC7 mice (Hines et al., 2006). Using the forced swim test (FST), female HET mice were shown to exhibit a significantly lesser time of being immobile during the duration of this test compared to the female WT mice (i.e., less “depression”). AC7 TG female mice were, on the other hand, found to exhibit longer periods of immobility than the female WT mice (more “depression”). There were no differences in the immobility time of male WT mice *versus* male TG, or male HET mice in the FST. In the tail suspension test (TST), female HET mice did not differ in immobility from WT mice, but the female TG mice showed greater periods of immobility. Again, there were no differences in immobility time in the TST between male HET, WT, and TG mice. These results indicate that in females, the overexpression of AC7 results in a higher level of “learned helplessness” (depressive-like behavior), while a reduction in AC7 expression produces greater resilience to depressive-like behaviors. A major caveat to this simple explanation, is the fact that the knock-down of AC7 in the HET female mice resulted in significant changes in gene expression of 30 other transcripts, and there were no changes in other transcript expression in the male mice. One of these transcripts in female strains, peroxiredoxin, has been implicated in behavior in the FST and TST (Scotton et al., 2020).

Table 1 summarizes the behavioral and physiological phenotypes elucidated in mice in which the expression of AC7 was manipulated.

### Genetic association of the ADCY7 gene with alcoholism and/or depression in humans

The results with genetic manipulation of AC7 expression in mice qualified this adenylyl cyclase as a possible candidate gene for a genetic contribution to human AUD (Boezio et al., 2017) and/or MDD. Desrivières et al. (2011) examined single nucleotide polymorphisms (SNPs) within the ADCY7 gene in humans for association with alcohol dependence (defined by DSM-IV and ICD-10 criteria). The subjects consisted of 1,703 individuals classified as alcohol dependent and 1,347 controls, and both men and women were included in this Caucasian population. A SNP (rs2302717) that defined a haplotype across a portion of AC7 gene (ADCY7) was found to be associated with alcohol dependence, but this association was only significant in the females. The minor allele (T) at this locus reduced the risk to develop alcohol dependence (OR = 0.71). Desrivières et al. (2011) noted that the haplotype identified by this SNP extended into the promoter region of ADCY7, and performed an analysis of the quantity of RNA for AC7 that was present in whole blood or adipose tissue from another large sample of Caucasian individuals. These studies revealed that the minor allele of rs2302717 correlated with lower *ADCY7* expression in both tissues. This was seemingly at odds with the data from the studies with mice (Desrivières et al., 2011), in which knock-down of Adcy7 and resultant diminution of AC7 RNA in brains of females resulted in less aversion to drinking, while in humans, a polymorphism that was protective against alcohol dependence was also responsible for lower levels of AC7 RNA. An obvious caveat is that the RNA measures in the HET mice were made in brain tissue while the human AC7 RNA was measured in blood and adipose tissues (Desrivières et al., 2011). The other caveat, already mentioned above, is the fact that the knock-down of Adcy7 in female mice results in changes in expression of a number of other transcripts in brains of the HET female mice, and this phenomenon will have to be explored in future studies, possibly in postmortem tissue of humans.

Given the finding that an allele that is protective against alcohol dependence in women, is also associated with lower levels of AC7 RNA in human blood, it is instructive to review a number of studies which measured adenylyl cyclase activity in human platelets and lymphocytes of alcoholics and control (non-alcoholic) subjects. The first of such studies (Tabakoff et al., 1988) included 95 alcoholic subjects and 33 controls, and the majority of the alcoholic subjects (all except 5 who had been abstinent by self-report for 12–48 months) were abstinent for 23 ± 16 days. All of the subjects were male. Measures of platelet adenylyl cyclase activity demonstrated no differences in basal activity, but significant differences between alcoholic and control subjects in cesium fluoride-, Gpp (NH)p-, and prostaglandin 1 (PGE1)-stimulated adenylyl cyclase activity, with the alcoholic subjects having lower stimulated adenylyl cyclase activity. At the time of this study, the various isoforms of adenylyl cyclase had not yet been described, but currently, it is known that AC7 is the dominant form of adenylyl cyclase in both platelets and lymphocytes (Hellevuo et al., 1993; Duan et al., 2010). Given the earlier discussion regarding the role of PKCδ in promoting the activation of AC7 by Gs protein, the presence of PKCδ and its significant physiological function in platelets, and the fact that all stimulatory agents used in the study of Tabakoff et al. (1988), were acting *via* the Gs protein to activate adenylyl cyclase, one cannot distinguish the effects as being related to upstream effects involving PKCδ, or to adenylyl cyclase *per se*. An additional observation made in this study, was that the five alcoholic individuals who had abstained for over 12 months, still displayed lower cesium fluoride-stimulated adenylyl cyclase activity. This led to the suggestion that the stimulated adenylyl cyclase activity in platelets may be a “trait” rather than a “state” marker in alcoholism (Tabakoff et al., 1988).

A different conclusion regarding adenylyl cyclase activity measured in lymphocytes of alcoholic and control subjects was provided by Szegedi et al. (1998). These investigators followed the adenylyl cyclase activity in lymphocytes of 73 alcohol-dependent subjects at admission to the clinic while intoxicated, at the time of maximal withdrawal signs, and after detoxification. Lymphocyte adenylyl cyclase activity of the alcohol-dependent subjects was also compared to control subjects. Their findings indicated that there were no differences in lymphocyte adenylyl cyclase activity between the control subjects and the alcohol-dependent subjects at admission, while the dependent subjects were intoxicated, but 2 days later basal, GTPγS-stimulated, and forskolin-stimulated adenylyl cyclase activity were significantly lower in the alcohol-dependent subjects going through withdrawal. After the withdrawal period, there again was no difference in adenylyl cyclase activity in lymphocytes of the control and alcohol-dependent subjects. The time course of changes in lymphocyte adenylyl cyclase activity in the studies of Szegedi et al. (1998) with humans, mirror the changes described in the striatum (Tabakoff and Hoffman, 1979) and cerebral cortex (Saito et al., 1987) of groups of mice chronically fed ethanol, during the early withdrawal period, and also several days after withdrawal. The earliest publication to note the differences in adenylyl cyclase activity (decreased adenosine (A_2_) receptor-mediated cyclic AMP production) in lymphocytes was that of Diamond et al. (1987). The alcohol-dependent subjects in that study were individuals described as “actively drinking” but having little or no alcohol in blood when blood was taken for isolation of lymphocytes. Thus, these subjects would resemble the “withdrawal” group in the studies of Szegedi et al. (1998). The changes described by Diamond et al. (1987) were evident in both B and T cells in the lymphocyte fraction, and more recent evidence examining the isoform of adenylyl cyclase in T and B cells, as well as macrophages, has identified the major adenylyl cyclase in these cells to be AC7 (Duan et al., 2010). Overall, the measurement of AC7 in lymphocytes may be advantageous for extrapolating to the activity of AC7 in brain of individuals undergoing withdrawal from chronic heavy alcohol consumption. It should be noted that the lower levels of adenylyl cyclase activity in platelets of alcoholics may also be a result of lowering of AC7 expression. But this change of expression would have to take place in the megakaryocytes, which are the precursors of platelets, since platelets do not contain DNA. Thus, at the least, the platelet measures of adenylyl cyclase activity would follow a time course more related to the time course of platelet turnover in blood, rather than a time course for changes in AC7 expression in cells replete with DNA and expression/translation machinery.

The platelet adenylyl cyclase activity measured in alcoholics may be confounded by other variables, particularly by the presence of comorbid MDD (Hoffman et al., 2002). In fact, the platelet adenylyl cyclase activity may be a trait marker for MDD which is in turn confounded by current alcohol use by the depressed subject (Hines and Tabakoff, 2005). The initial studies of platelet adenylyl cyclase activity in depressed subjects indicated that forskolin-stimulated adenylyl cyclase activity was particularly lower in individuals diagnosed with MDD, compared to control subjects (Menninger and Tabakoff, 1997). Since forskolin acts directly on the adenylyl cyclase protein to enhance activity (Seamon et al., 1981), one can surmise that depressed subjects have reduced quantities of adenylyl cyclase protein in platelets, and since there is evidence that the major form of adenylyl cyclase in platelets is AC7, one can go further to consider that depressed subjects have lower levels of AC7 in platelets. This supposition was strengthened by the work of Hines et al. (2006), which also proposed an explanation for the lower levels of AC7 in platelets of humans suffering from depression. Hellevuo et al. (1997) demonstrated that the AC7 gene in humans is characterized by a series of polymorphic repeats in the 3′-UTR. The findings of Hines et al. (2006) indicated that the lowest levels of forskolin-stimulated adenylyl cyclase activity in platelets were in depressed subjects whose DNA in the 3′-UTR harbored the longest stretch (seven repeats) of the tetranucleotide AACA (Hellevuo et al., 1997). Some other observations generated by the work of Hines et al. (2006) were: the most prominent diminution in forskolin-stimulated platelet adenylyl cyclase activity was noted in depressed subjects who also had a family history of depressive illness; females diagnosed with MDD with a family history of depression; and in individuals having a genotype for the seven repeats of AACA.

Through a combination of studies on gene expression and informatics using AC7 TG and WT mice, AC7 was linked to function of the proopiomelanocortin (POMC) system and immune system function. Clearly there is a link between the POMC transcript, stress, and the immune system, since POMC is the precursor to ACTH, and pituitary ACTH release, instigated by CRF, stimulates release of adrenal glucocorticoids, and modulates the activity of the immune system (Leistner and Menke, 2018). Chronic stress, in conjunction with childhood trauma, has been considered a significant contributory factor to the development of major depression (Heim and Nemeroff, 2001). Although the relationship of stress and depression has been considered to arise *via* the activation of corticosteroid receptors in brain (Holsboer, 2000), with polymorphisms in FKBP5 (a co-chaperone for the glucocorticoid receptor) being an important component of this relationship (Binder et al., 2004), the above described function of AC7 in control of CRF-mediated ACTH release (Antoni et al., 2003; Pronko et al., 2010) should also be considered in the etiology of depression. Furthermore, it is now becoming evident that AC7 is the major form of adenylyl cyclase in the immune system, and controls activation of macrophages, as well as B and T lymphocytes (Jiang et al., 2008; Duan et al., 2010). Recent studies also indicate that AC7 is the major form of adenylyl cyclase expressed in mouse and human microglia (Bennett et al., 2016; Galatro et al., 2017). Microglia are considered the “macrophages” of the CNS, and it is not surprising that AC7 is expressed in microglia. (Table 2 shows the expression levels of the various isoforms of adenylyl cyclase in microglia). Cyclic AMP levels are important for conversion of microglia from the M1 to M2 phenotype (M1 describes a proinflammatory phenotype, and M2 an activated but reparatory phenotype) (Ghosh et al., 2016). The relationship of stress to microglial activation is well summarized in Yirmiya et al. (2015) and these authors propose that some forms of MDD may be a “microglial disease,” dependent on microglia transitioning to the M1 phenotype. In all, the involvement of AC7 in the CRF-mediated release of ACTH from the pituitary, and involvement in microglia activation status, may play an important role in the etiology of depression. Assuming that AC7 is mediating the CRF-stimulated ACTH release, and the stress response is of consequence in the etiology of MDD, the sex differences described earlier in the ACTH and glucocorticoid responses in the WT mice, *versus* those with genetically modified expression of AC7, are notable (Pronko et al., 2010).

**TABLE 2:** Microglia were isolated at autopsy from parietal lcortex of 39 human subjects. RNASeq was performed on total RNA extracted from flow cytometry-sorted cells. Values represent median microglia expression levels (RPKM, reads per kilobase of transcript per million reads mapped). Galatro et al., 2017.

Expression levels of adenylyl cyclase	Isoforms in microglia
AC isoform	RPKM
ADCY1	0.071
ADCY2	0.038
ADCY3	1.867
ADCY4	0.619
ADCY5	0.023
ADCY6	2.073
ADCY7	52.570
ADCY8	0
ADCY9	1.219
ADCY10	0.223

Further evidence for the involvement of AC7 in depression emanated from the laboratories of Etienne Sibille (Joeyen-Waldorf et al., 2012). This group used mice lacking the serotonin transporter (SERT^KO^), which have been considered to be a model for studying depressive behaviors and emotionality (Lira et al., 2003), to assess gene expression in amygdala and cingulate cortex. They then compared the differentially expressed transcripts noted between the SERT^KO^ and WT mice to differentially expressed transcripts noted in postmortem samples of amygdala and cingulate cortex from humans with familial MDD, and matched controls. “Conserved changes” were found for 31 transcripts in the amygdala, and 20 transcripts in cingulate cortex in comparisons of the mouse and human brain samples, and the transcript for AC7 was found to be significantly upregulated in the brain tissue from the SERT^KO^ mice, compared to the WT controls, and in the brain tissue of depressed subjects compared to their matched controls. Their further studies examined (using BOLD (MRI)) threat-related amygdala reactivity in two independent samples of human subjects and its association with a single nucleotide polymorphism in the ADCY7 gene. This SNP (rs1064448) has previously been shown to identify a haplotype including a major portion of the ADCY7 and the 3′UTR containing the tetranucleotide repeats (Hines et al., 2006). In both samples, there was a significant association of rs1064448 with greater threat-related amygdala reactivity (Joeyen-Waldorf et al., 2012). These studies illustrate the possible importance of ADCY7 in fear-related amygdala function, and “conserved changes” in the expression of AC7 transcript in brain tissue from mice used as a model of depressive behavior (Joeyen-Waldorf et al., 2012), and in human subjects diagnosed with MDD, amplify the studies of Hines, et al. (2006). The SERT^KO^ mice exhibited higher levels of expression of AC7 in brain tissue, as did the post-mortem tissue of the depressed human subjects, and in the studies of Hines et al. (2006), it was the genetically manipulated mice with lower levels of AC7 in brain that exhibited the lesser depressive-like behavior in the FST, and the female animals with the higher expression of AC7 showed higher immobility in the FST. It is parsimonious to think that deletion or pharmacologic blockade of SERT is coupled to upregulation of AC7 RNA, but this implication of the relationship of SERT and AC7 expression in development or treatment of depression does not appear straightforward. One has to be careful in making generalizations from the results obtained with the SERT^KO^ mice produced on the 129/SvEv genetic background since the same genetic manipulation produced no effect in the C57BL/6 mice (Lira et al., 2003).

The effect of antidepressants on the activity and possibly the expression of adenylyl cyclase in brain may or may not be reflected in measures of adenylyl cyclase activity in platelets since platelet adenylyl cyclase activity was found to be lower in depressed subjects compared to controls (Hines et al., 2006). A more recent study of platelet adenylyl cyclase activity stimulated by PGE1 (*via* the GS-coupled prostaglandin EP1 receptor) also demonstrated that subjects diagnosed with MDD had significantly lower PGE1-stimulated platelet adenylyl cyclase activity than control subjects (Targum et al., 2022). This study, however, followed a subset of subjects through a 6-week period of treatment with antidepressants which were primarily inhibitors of SERT (SSRIs). In the subjects that showed significant improvement in their Hamilton Depression Ratings (Ham D_17_ and Ham D_6_), there was also a significant increase in their PGE1-stimulated adenylyl cyclase activity toward levels measured in control subjects. Targum et al. (2022), therefore, replicated the lower adenylyl cyclase activity in platelets of clinically depressed subjects (Hines et al., 2006), but also added the fact that the platelet adenylyl cyclase can be not only a marker for depression, but also for measuring response to antidepressants. Targum et al. (2022) also proposed a mechanism for their observed results to be the sequestration of the Gs protein in lipid rafts (Allen et al., 2009) in the platelets of the depressed subjects and suggested that antidepressant treatment would result in the release of Gs from sequestration to be available for stimulation of adenylyl cyclase. Unfortunately, forskolin-stimulated adenylyl cyclase activity was not measured in the studies of Targum et al. (2022) to distinguish between the proposed mechanism, and the diminution of the adenylyl cyclase protein as proposed in other studies (Hines et al., 2006). There may well be different mechanisms in play which result in higher levels of the RNA for AC7 in brain in conjunction with signs of depression in genetically manipulated mice, and depressed human subjects (Hines et al., 2006; Joeyen-Waldorf et al., 2012), and the lower levels of adenylyl cyclase activity (presumably AC7), activated by various means, in platelets of depressed humans. Although forskolin can enhance adenylyl cyclase activity independent of other factors (Seamon et al., 1981), and forskolin (radioactively labeled), can be used to quantify adenylyl cyclase protein (Insel and Ostrom, 2003), there is clear evidence that Gsα can further activate adenylyl cyclase catalytic function (Insel and Ostrom, 2003). Thus, the lower levels of adenylyl cyclase activity, stimulated by forskolin or agents acting *via* Gs proteins, in platelets of depressed subjects may be either a result of lower levels of the adenylyl cyclase protein, a sequestration of Gs protein, or both mechanisms. Whether the proposed mechanism involving the sequestration of Gs in platelets (Targum et al., 2022) in depressed subjects extends to brain (Senese and Rasenick, 2021), bears scrutiny.

## Summary and consideration of AC7 as a therapeutic target to treat alcoholism and/or depression

AC7 is a member of the sub-family of adenylyl cyclases (Type 2, 4, and 7) whose activity is insensitive to Giα proteins, is potentiated by the βγ subunits of G proteins in conjunction with Gsα stimulation, and whose responsiveness to Gsα is modulated by the state of phosphorylation catalyzed by PKCδ. This enzyme is also insensitive to calcium in the presence or absence of calmodulin. The distinguishing feature that separates AC7 from the Type 2 and Type 4 adenylyl cyclases is the particularly high level of activation of this enzyme by ethanol when the enzyme activity is also influenced by Gsα. AC7 also has a cellular/tissue distribution that distinguishes it from the other members of its sub-family. Particularly notable is the evidence for its presence in the amygdala, nucleus accumbens, hippocampus, and frontal cortical regions in brains of animals, with evidence for presynaptic and postsynaptic localization (Mons et al., 1998), and its presence in the corticotrophs of the pituitary. The presence of AC7 in the pituitary, and its involvement in the release of ACTH, speaks to the possible importance of AC7 in the hypothalamic/pituitary/adrenocortical response to stress. The presence of AC7 in the amygdala, and possibly in other parts of the striatum, as well as in frontal cortical regions, and its coupling to the CRF1 receptor in the amygdala, as well as in the pituitary, bespeaks a deeper involvement in stress and negative affect. It is, thus, not surprising that associations have been reported between measures of adenylyl cyclase activity in brain, platelets, and lymphocytes of alcoholics, and subjects diagnosed with MDD. This association has been extended to genetic markers which identify the haplotype in which the ADCY7 gene is located.

The significant comorbidity that exists between AUD and MDD is well accepted (Hasin et al., 2018). There are two manifestations of the co-occurrence of depression in individuals who fit the criteria for AUD. In one manifestation, the signs of depression are evident only during the initial period of time that an individual dependent on alcohol tried to abstain (i.e., alcohol withdrawal), and once abstinence has been achieved for some period of time, the signs and symptoms of depression abate (Raimo and Schuckit, 1998). In another manifestation, the signs of depression become evident during the initial stages of abstinence but continue to persist throughout sobriety (Raimo and Schuckit, 1998). When one considers the time course of changes in brains of animals that have been chronically fed ethanol, and have undergone forced abstinence, one notes that in brain areas such as the cortex, the activity of adenylyl cyclase is within the normal range while the animal is intoxicated, drops below normal levels during the first days of abstinence, and then returns to normal. One wonders whether the lower levels of adenylyl cyclase activity in brain during the initial stages of withdrawal is a contributing factor to the signs of withdrawal (i.e., depression), or simply a byproduct of the withdrawal hyperexcitability syndrome. It is of interest that measures of adenylyl cyclase in platelets of human alcoholics present a picture resembling the time course of fluctuations in adenylyl cyclase activity seen in brains of alcohol dependent and withdrawing animals. Adenylyl cyclase activity in platelets was in the normal range while the individual was actively consuming alcohol, dropped below normal levels during early stages of withdrawal, and then returned to normal after a period of abstinence. It might seem that the stress of abstaining from alcohol may be a factor in diminishing adenylyl cyclase activity during withdrawal. The alcohol withdrawal-induced changes in brain and platelet adenylyl cyclase activity can be classified as a state marker of withdrawal from chronic use of alcohol.

On the other hand, the genetically generated increased expression of adenylyl cyclase in brains of animals is associated with more permanent depressive symptomology. There are a number of missing pieces of evidence that need to be added to assume that increases in mRNA for AC7 are related to higher activity of this enzyme in brain of depressed subjects. Even accurate measures of AC7 protein have not been accomplished (Joeyen-Waldorf et al., 2012).

The differences in adenylyl cyclase activity between depressed human subjects and controls, are related to lower levels of adenylyl cyclase activity in depressed subjects in platelets, and activity of this adenylyl cyclase in response to Gsα is enhanced when the subject is being successfully treated with antidepressants (Targum et al., 2022). Even though the exact relationship between expression and activity of AC7 and MDD is still enigmatic, the development of pharmacological tools for isoform-selective manipulation of AC7 would help resolve the enigmatic features of the relationship and may lead to novel therapeutics for depression and/or AUD.

### AC7 as a therapeutic target

A prior review (Price and Brust, 2019) suggested the possibility that AC7 may be “A new target for depression,” but did not propose how to “medicate this target.” An excellent review of molecules that inhibit adenylyl cyclase activity is available (Seifert et al., 2012) including many P-site inhibitors (adenosine analogues) and substituted nucleotides that act at the catalytic site of the adenylyl cyclases. This review emphasizes the problems encountered in trying to generate inhibitors that interact with the catalytic domains of the adenylyl cyclases, and would also have some substantial selectivity among the nine membrane-bound adenylyl cyclases, and would not have off-target effects on ion channels and glucose transporters. The effects of the large number of compounds developed as catalytic site inhibitors have not been tested for activity and selectivity for AC7. The diterpene alkaloids, such as forskolin, have generally been thought of as activators of adenylyl cyclase (Seamon et al., 1981), however, 1-deoxy-forskolin and 1,9-dideoxy-forskolin have been found to inhibit adenylyl cyclase activity (Seifert et al., 2012). One of the most interesting analogues of forskolin is referred to as BODIPY-FS (Pinto et al., 2008). BODIPY-FS is an activator of Type 1 and 5 adenylyl cyclases, has little effect on Type 3 and 6, but inhibits the activity of the Type 2 adenylyl cyclase (Seifert et al., 2012). The other members of the Type 2 adenylyl cyclase family, i.e., Type 4 and Type 7, have not been explored with regard to their responses to BODIPY-FS.

The search for isoform-specific inhibitors (Brand et al., 2013) or activators of adenylyl cyclases may benefit from a more thorough consideration of structural/regulatory differences. In this regard, peptide analogues designed to correspond to the regions of the Type 2, 4 and 7 adenylyl cyclases that are phosphorylated by PKC may prove valuable modulators of these isoforms, and may even distinguish between these isoforms. Interestingly, a peptide based on the C1b region of AC7 was found to be an inhibitor of this enzyme (Yan et al., 2001). The other sequence that bears attention in trying to modulate the activity of AC7 is the region that binds the β/γ subunits (Diel et al., 2006; Brand, 2015). The sequences in this region of the isoforms responsive to β/γ need to be carefully examined and information should be gathered on the variants of the β and γ subunits that may have selectivity for the particular cyclase isoforms Diel et al. (2006). Such peptide modulators would have an additional advantage (or possible disadvantage) of being coordinate regulators of, for example, AC7 requiring the binding of Gsα, for evidence of their activity (Wang et al., 2005). Development of peptides as drug molecules is a complicated endeavor and as mentioned above, targeting AC7 should be preceded by a clear knowledge of its role in brain function [e.g. opiate tolerance/dependence (Wang et al., 2005), AUD, depression, etc.] as well as in the periphery [e.g. in the immune system (Duan et al., 2010)]. At this point, the evidence for AC7 activity in depression and AUD is tantalizing, but far from definitive. The actions of lithium (a mood stabilizer) as an inhibitor of AC7 (Mann et al., 2008), and the possible upregulation of AC7 by antidepressants such as SSRIs (Targum et al., 2022) should raise interest in the role of this enzyme in mood disorders. On the other hand, the more immediate use of AC7 activity may be as a peripheral state marker in AUD, state or trait marker in depression and a diagnostic distinguishing MDD from AUD (Tabakoff et al., 1986) or manic-depressive illness (Tabakoff et al., 2010).
